# Healing comparison of porcine cutaneous incisions made with cold steel scalpel, standard electrosurgical blade, and a novel tissue dissector

**DOI:** 10.15761/MRI.1000124

**Published:** 2017-10-27

**Authors:** Albert Y Wu, Thomas J Baldwin, Bhupendra C Patel, Jeffrey W Clymer, Ryan D Lewis

**Affiliations:** 1 Stanford University Medical Center, USA; 2 Utah Veterinary Diagnostic Laboratory, Utah State University, USA; 3 School of Medicine, University of Utah, Salt Lake City, USA; 4 Ethicon, Inc., Cincinnati, USA

**Keywords:** electrosurgery, thermal damage, wound healing, cosmesis, porcine

## Abstract

**Background:**

Standard electrosurgery provides superior hemostasis compared to a cold steel scalpel, but inferior tissue healing. A novel electrosurgical blade with an advanced waveform, the MEGADYNE ACE BLADE™ 700 Soft Tissue Dissector (ACE), was designed to provide both excellent hemostasis and wound healing. This study compared ACE to scalpel and standard electrosurgery in a porcine model of wound healing.

**Methods:**

Skin incisions from six pigs were evaluated at time points of 0, 1, 2, 3 and 6 weeks after application of the three devices. Histopathology was performed on samples from each time point. For each non-initial time point, the healing incisions were photographed for later evaluation by expert graders, and excised for wound strength testing.

**Results:**

Time 0 photomicrographs showed a gradient of thermal tissue damage by initial incision, ranging from no damage made by the scalpel, minimal damage made by ACE, and twice the ACE damage made by a nonstick PTFE-coated electrosurgical blade. Histopathologic analysis at 6 weeks showed comparable dermal scar width measurements for scalpel and ACE incisions. Scars were wider for incisions made by standard electrosurgical blade. Wound strength was greater for scalpel and ACE than for standard electrosurgery. Cosmetic results at 6 weeks were not significantly different between scalpel and ACE incisions, while standard electrosurgical blade incisions were significantly inferior to ACE (odds ratio: 53.4, p<0.001).

**Conclusion:**

The MEGADYNE ACE BLADE™ 700 Soft Tissue Dissector represents a significant improvement in electrosurgical technology for skin incisions and dispels the traditional concerns of delayed healing and poor cosmetic result that have been attributed to using conventional electrosurgical blades for skin incisions.

## Introduction

The scalpel or some variation of it has been present throughout the history of surgical practice. While the scalpel is likely to serve surgeons well into the future, it has its drawbacks. From a safety perspective, the scalpel poses the risk of unintended injury in the operating room. With regard to durability, the micro-fine cutting edge dulls rapidly, especially when used in tough tissue and can require several blades to be used during a single procedure. From a utility standpoint, the scalpel can only cut and has no other function; therefore, when the cutting is accomplished the scalpel must be handed off the field, which consumes time and increases the risk of scalpel injuries. Additionally, the surgeon often has to keep the skin on stretch to counter the drag produced by the scalpel to prevent scalloping or “beveling” of the skin edges [1].

Several devices have provided cutting ability in applications where a scalpel would normally be used, but those instruments have disadvantages and do not provide “skin-to-skin” cutting utility. Lasers, for example, are useful at cutting where coagulation is desired, but because of the collateral tissue damage, they are not recommended for primary skin incisions when cosmetic results are important [2,3]. Conventional electrosurgery has similar shortcomings and a number of studies have shown delayed wound healing attributable to thermal damage [4-8]. The MEGADYNE ACE BLADE™ 700 Soft Tissue Dissector (Ethicon, Inc., Cincinnati, OH) is a novel electrosurgical blade that has been geometrically tuned to work in tandem with the proprietary, constant voltage-based ACE Mode found in MegaPower^®^ electrosurgical generators. The combination of unique blade geometry and the special ACE waveform provides an instrument that easily cuts through tissue, including skin, while causing significantly less thermal damage to collateral tissues when compared to conventional electrosurgical blades.

**Figure 1.**
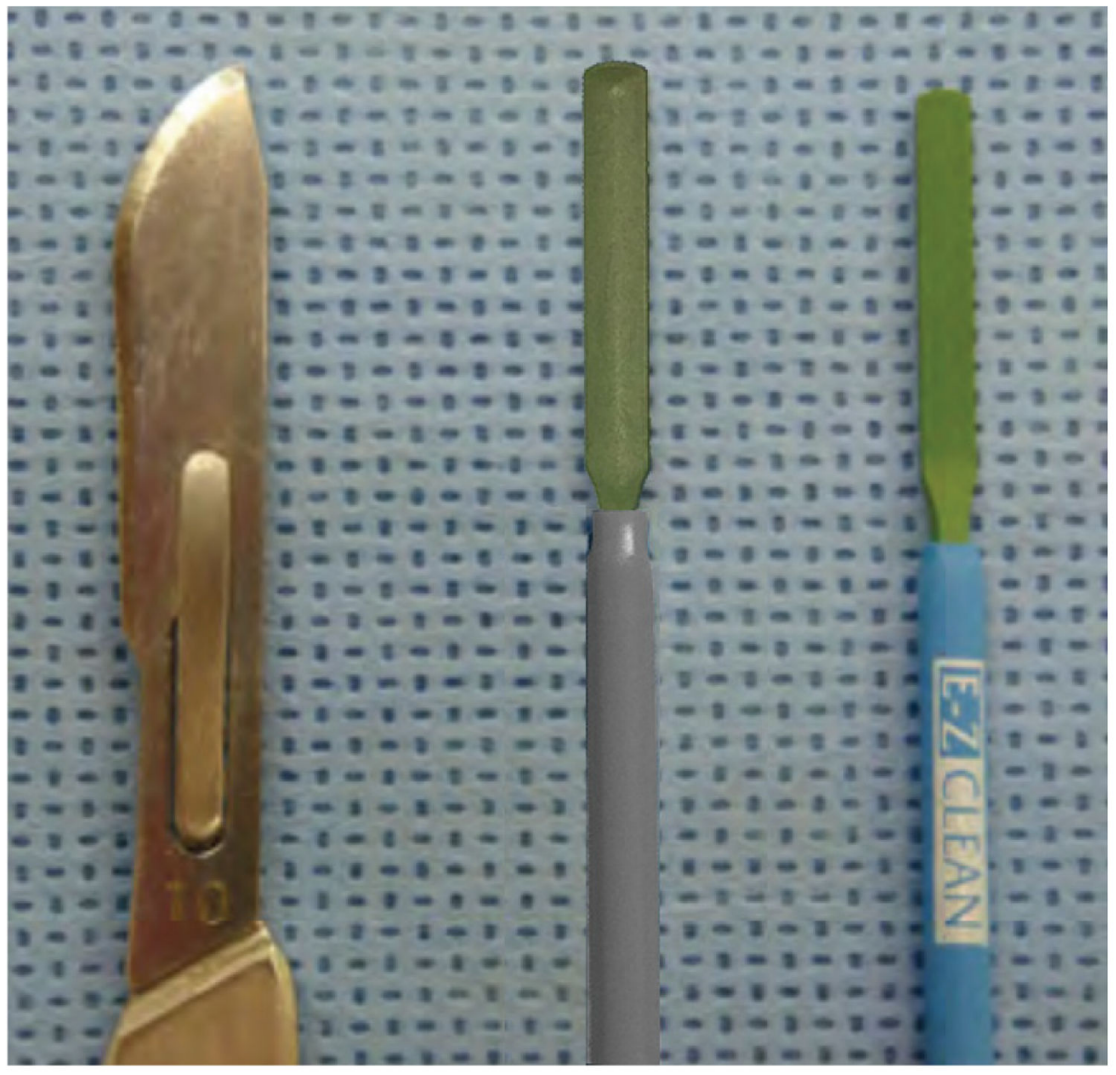
Photograph of cold steel scalpel (left), ACE Blade with unique geometry (middle), and standard PTFE coated electrosurgical blade (right).

This study was undertaken to demonstrate the effectiveness of ACE for making skin incisions and compares those incisions in strength, histological appearance and cosmetic result to incisions made with a cold steel scalpel and standard electrosurgical blade (Figure 1).

## Materials and methods

### Animal model

Six live domestic female pigs weighing an average of 35 kg were acquired as test subjects. All aspects of the protocol were carried out in accordance with requirements of the Animal Welfare Act and the National Research Council guidance for the care and use of laboratory animals. The protocol was reviewed and approved by the study facility’s Institutional Animal Care and Use Committee, institutional review board, veterinary pathologist, independent investigators, and the study sponsor.

### Surgical procedure

Individual animals were induced with tiletamine/zolazepam (4.4 mg/kg) and xylazine (2.2 mg/kg) administered intramuscularly prior to intubation. General anesthesia was attained using 0.5% to 5.0% isoflurane in oxygen according to effect. Post-operative analgesia was provided by administering buprenorphine (0.005–0.02 mg/kg) intramuscularly. Each animal was prepared for aseptic surgery.

**Figure 2.**
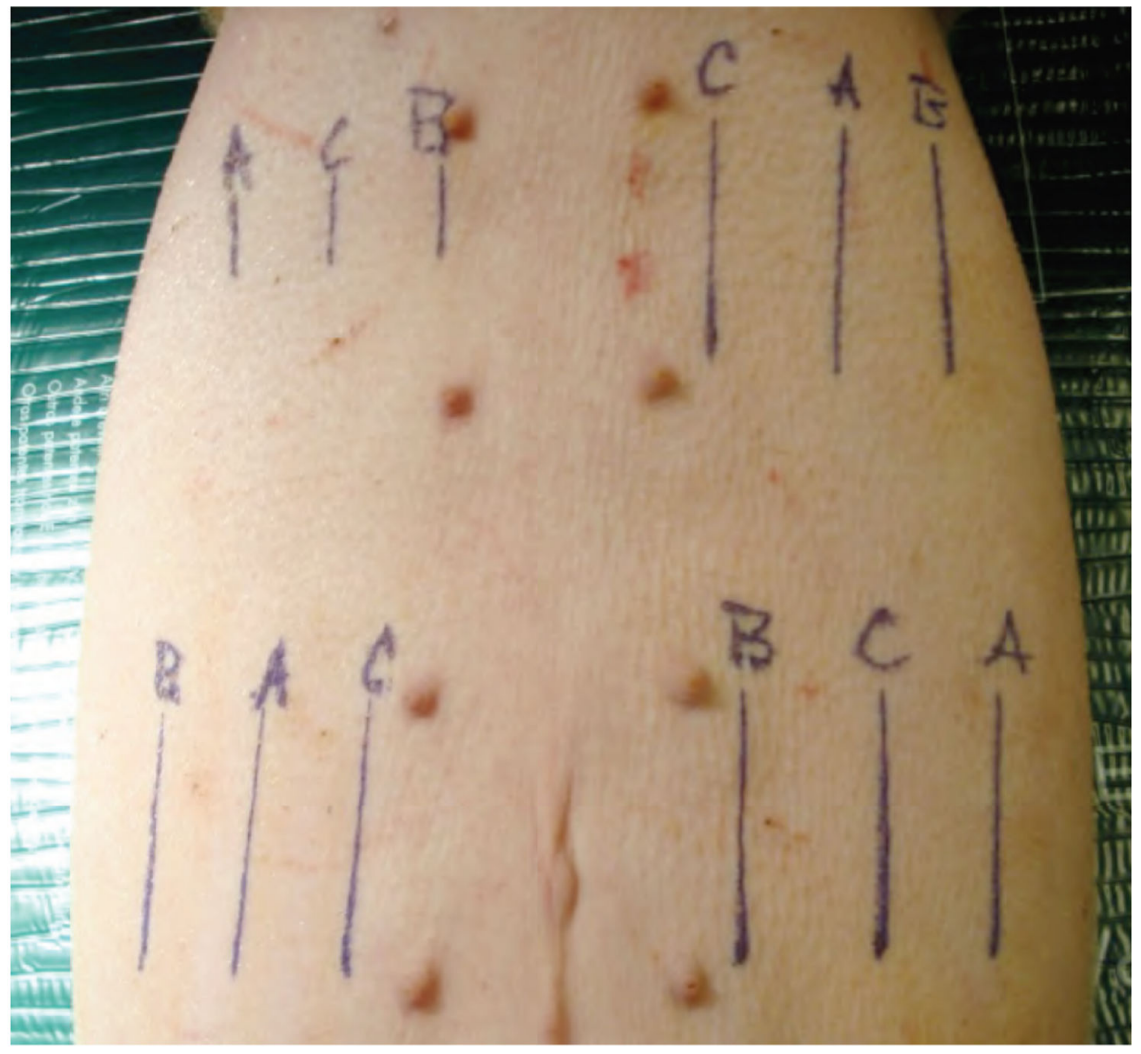
Pre-operative markings showing distribution and orientation of incision types. A=ACE; B=Scalpel blade; C=Standard electrosurgical blade. The three 3 cm incisions (right upper quadrant) are for histological analysis. The remaining 6 cm incisions were used for wound strength testing.

The abdomen of each pig was divided into quadrants and incisions were varied in a predetermined random pattern to eliminate potential location bias (Figure 2). A template was used to mark lines in each of the quadrants to aid in uniform spacing and length of the incisions. Twelve incisions were made in the ventral abdominal skin, spaced to minimize vascular compromise. Incisions were made through the epidermis, dermis, and approximately 1 cm into the subcutis. Scalpel incisions were made with one stroke where possible. ACE incisions were made in ACE Mode (constant voltage) and standard electrosurgical cut incisions were made in constant power cut mode at 40 watts using a commercially available PTFE non-stick electrosurgical blade. Hemostasis of transected blood vessels was achieved using the “coag” mode on the MegaPower electrosurgical generator set at 30 watts. Three 6 cm long incisions for wound strength testing were made in three of the quadrants and three 3 cm long incisions were made in one of the quadrants for histopathology.

One animal was selected as the T-0 specimen and the 3 cm abdominal incisions were harvested and placed immediately in 10% neutral buffered formalin. The T-0 animal was then euthanized via injection of a concentrated barbiturate solution.

All wounds on the remaining 5 pigs were closed in two layers, with the subcutaneous layer closed using 4-0 VICRYL (Ethicon, Inc. Somerville, NJ) and the epidermis with 3-0 Monolon monofilament nylon suture (VEDCO Products, St. Joseph, MO). The subcutis was closed with interrupted sutures. The epidermis was closed in a simple continuous pattern. Wounds were dressed by spreading Povidoneiodine Ointment (VEDCO Products, St. Joseph, MO) over the length of the incisions to prevent infection. Partially and healed incisions were excised at 1, 2, 3, and 6 weeks post incisions and placed in 10% neutral buffered formalin.

### Wound evaluation time points

For the first week post procedure, animals were observed for evidence of infection, suture line dehiscence, and trauma. Sutures were removed from all animals (except the time 0 and 1 week animals) at 2 weeks to ensure wound integrity. Individual animals were survived to 1 week, 2 weeks, 3 weeks and 6 weeks respectively, with one animal reserved as an alternate. Sutures were removed from the week 1 samples just prior to pull testing.

Incision sites were photographed at each time point prior to *en bloc* excision of samples from each quadrant. The samples were placed immediately in a 10% formalin solution. The 3-cm samples were reserved for histopathologic examination. The 6-cm samples were reserved for wound strength testing.

Histopathologic specimens for wound healing evaluation were fixed in 10% formalin for a minimum of 24 hours prior to processing. Representative cross sections for each sample at each time point were made using a standard paraffin embedding process. Slide mounted samples were stained using hematoxylin and eosin and Masson’s trichrome stain. Slides were examined by light microscopy for evidence of epidermal hyperplasia, surface inflammatory crust, severity and type of tissue inflammatory response as well as average width of dermal scar tissue. Four sections of each sample were averaged to produce a mean width of dermal scar tissue.

### Wound strength test methodology

Samples from the 1, 2, 3 and 6 week animals were tested for wound strength on an Instron^®^ Model 4464 Universal Testing Machine (Illinois Tool Works, Inc., Norwood, MA). Sample preparation involved removing harvested tissue blocks from 10% formalin solution within 24 hours after harvesting and placing them into a normal saline bath for 2 hours. Samples were then divided into 1 x 3 cm segments for testing. Four test samples were taken from each 6-cm wound for strength testing. Twelve samples per test type (scalpel, ACE, and standard electrosurgical blade) were pulled to failure for each time point of 1, 2, 3 and 6 weeks. Data was recorded as maximum pounds force (lbf) and the mean was determined for each sample type.

### Cosmetic healing evaluation methodology

Photographs were taken of the wound surface characteristics at 1, 2, 3 and 6 weeks and compiled for evaluation. A total of 12 plastic surgeons or plastic surgeon residents were shown photographs in a blinded fashion and asked to rank the healed incisions from best to worst. In each of the four zones, or quadrants, the surgeons looked at side-by-side incisions, one incision from each device. The instructions were to assess “visibility and quality of the scar” at the incision. The term “cosmetic healing” was not used in the study instructions, but added by the authors to simplify presentation and discussion of results. Statistical comparisons were performed via ordinal logistic regression with α=0.05.

## Results

### Histopathologic analysis

#### Week 0:

The scalpel incision showed no evidence of collateral cellular necrosis or vacuolation of the keratinocytes in the epidermal layer. Similarly, the dermal layer of the incisions made by scalpel showed no evidence of collagen degeneration, edema or early leukocyte infiltration or margination. The only evidence of collateral damage by scalpel incision was occasional small hemorrhages in the subcutis (Figure 3a). The ACE incision showed mild collateral cellular necrosis, although keratinocytes in the epidermal layer maintained cell membrane and nuclear structure. Similarly, the dermal layer of the incisions made by ACE showed moderate collagen degeneration and mild edema with no early leukocyte infiltration or margination (Figure 3b). Conventional electrosurgical blade incision also showed collateral cellular necrosis, but keratinocytes in the epidermal layer exhibited loss of cell membrane and nuclear structure. The dermal layer of the incisions made by the conventional electrosurgical cut showed severe collagen degeneration, moderate to severe edema and mild early leukocyte infiltration and margination (Figure 3c).

#### Week 1:

**Figure 4.**
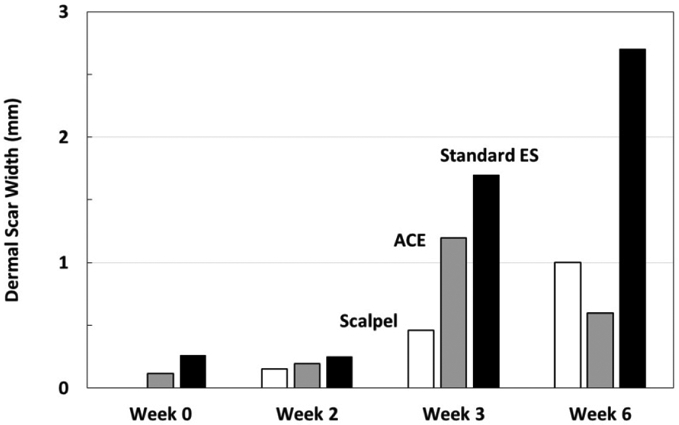
Average width of dermal scar as evidence of wound healing progression. Note at 2 and 3 weeks, the average dermal scar values for ACE incisions are greater than scalpel incisions. At 6 weeks, the ACE dermal scar widths and the scalpel widths are similar, while the standard electrosurgical cut values indicate a greater scar width.

The incision made by scalpel showed indications of complete re-epithelization of the epidermis while samples of incisions made by ACE and the conventional electrosurgical blade showed partial healing of the epidermis and some evidence of fibrin exudates. Dermal healing at week 1 was characterized by a mild granulomatous inflammatory response seen in the incision made by scalpel and moderate responses in both ACE and the standard electrosurgical blade. Evidence of thermal damage cellular degeneration, was observed in both ACE and standard electrosurgical blade samples, but not seen in the incision made by scalpel (Table 1) (Figure 4).

#### Week 2:

All samples demonstrated re-epithelialization at this point. Observation of the skin surface characteristics showed no surface inflammatory crust on the samples made by ACE, minimal crust on the sample made by scalpel and moderate crusting on the sample made by standard electrosurgical blade.

#### Week 3:

At 3 weeks, all epidermal surfaces were well healed.

#### Week 6:

At 6 weeks, all wounds were intact and grossly appeared to be well healed. Histopathology indicated a mild residual granulomatous inflammatory response for the incisions made using ACE. Incisions made using the standard electrosurgical blade also showed a mild granulomatous response but lymphocytic infiltration was noted also. Incisions made by scalpel had minimal granulomatous inflammatory response.

### Wound strength

**Figure 5.**
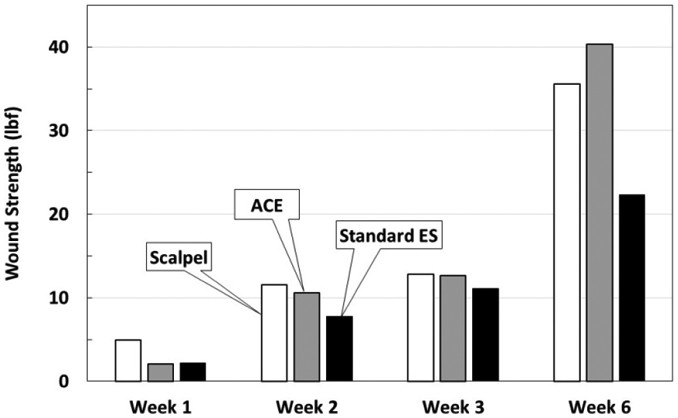
Wound strength testing measured in pounds force. Individual samples were pulled in force vectors perpendicular to the incision until failure. The values represent average lbf. Note at week 6, standard electrosurgical blade incisions are not as strong as ACE or scalpel incisions.

For each time point (1, 2, 3 and 6 weeks), 12 samples were tested for wound strength (Table 1) (Figure 5). By week 6, scalpel and ACE showed prominently higher wound strength than standard electrosurgical blade.

### Cosmetic wound healing results

**Figure 6.**
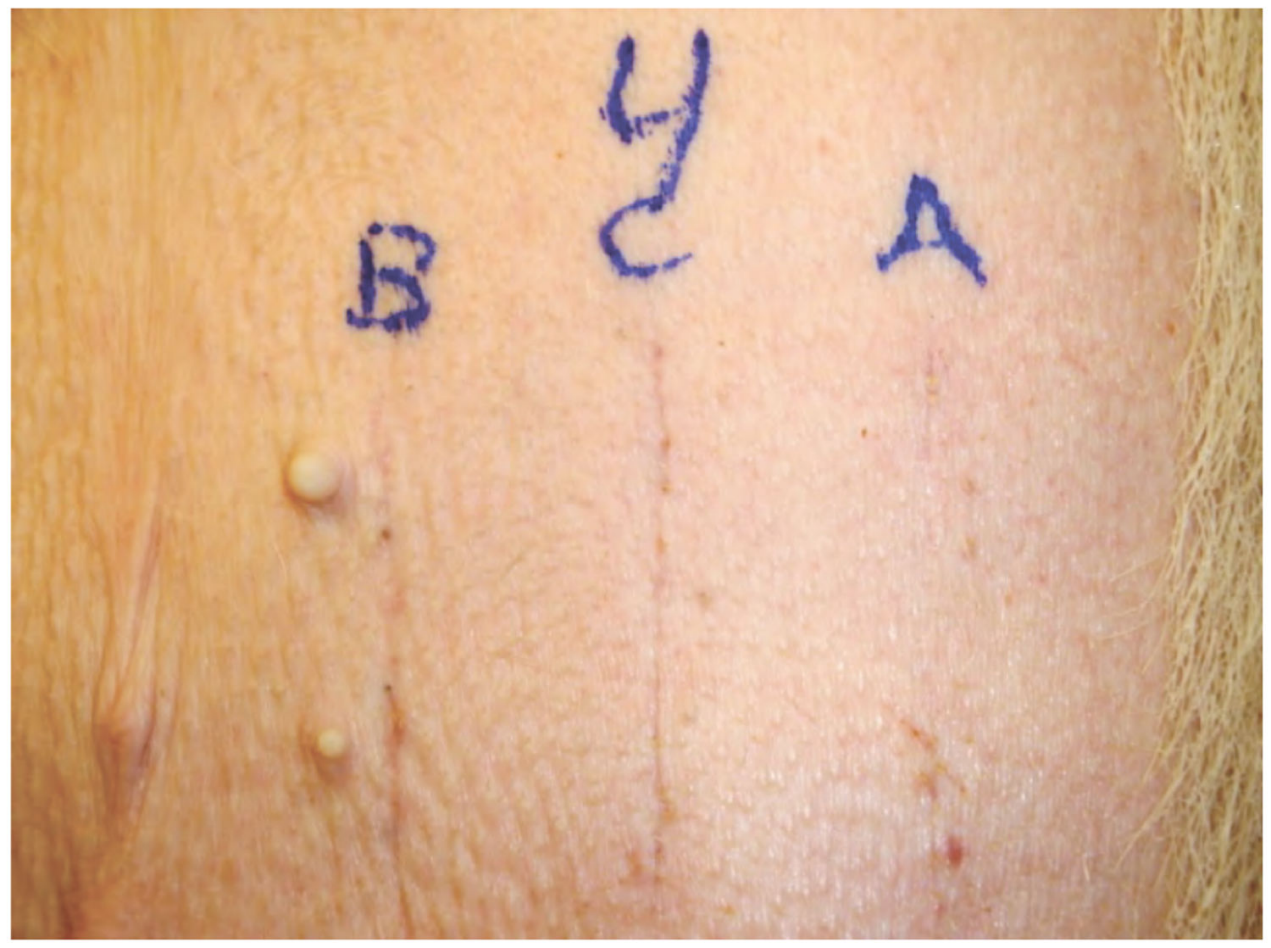
Photograph of 6 week surface healing from pig number 4, which survived to week 6. A=Incision made by ACE Blade in ACE Mode. B=Scalpel incision. C=Standard electrosurgical blade incision.

**Figure 7.**
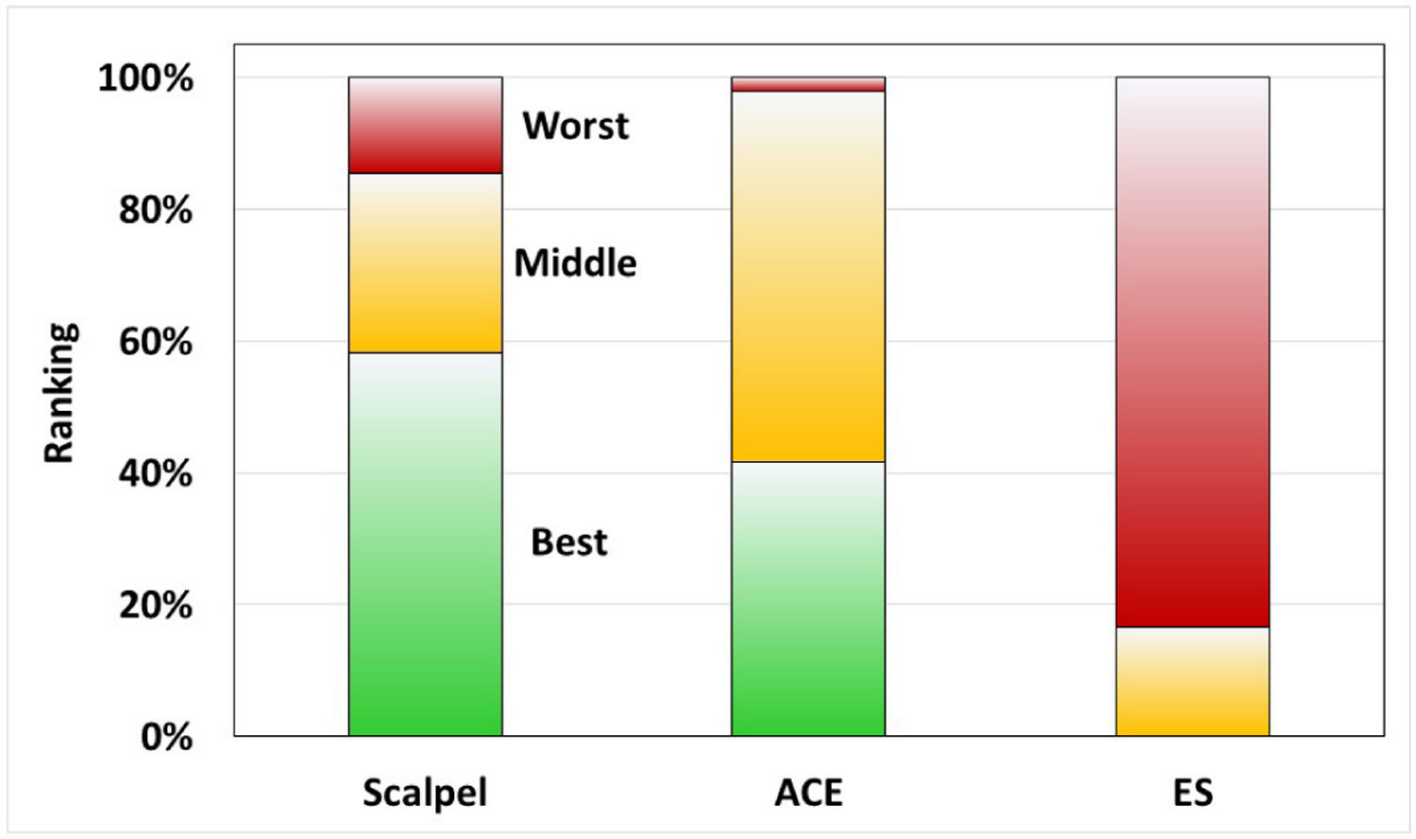
Ranked expert reports of incision healing at week 6.

Each of the 12 plastic surgeon evaluators blinded to the testing was given 4 photos of each pig representing the 3 randomized incisions created in each abdominal quadrant (Figure 6). Data from all 6 weeks were pooled for a total of 48 wound group observations. Out of 48 observations, scalpel incisions were ranked best 28 times, ACE incisions were ranked best 20 times and conventional electrosurgical blade incisions were ranked best 0 times (Figure 7). Via ordinal logistic regression, ACE was ranked significantly higher than standard electrosurgical blade (odds ratio: 53.4, p<0.001), but not significantly different from scalpel (p=0.381).

## Discussion

Wound healing trajectory studies have demonstrated a more rapid return to normal structure and appearance for skin incisions when less tissue damage is present initially [9-11]. In this study, histopathology revealed differences between the three incision types at time 0. As anticipated, the scalpel incision caused mechanical damage to the epidermal, dermal and subcuticular layers. There was no evidence of thermal damage such as vacuolation, collagen degeneration, or edema. Both ACE and standard electrosurgical blade showed evidence of thermal damage to the tissues at time 0. The standard electrosurgical blade resulted in approximately two times greater damage than ACE.

Comparison of the time 0 histology would predict that scalpel wounds (least initial tissue damage) would heal the fastest, followed by ACE incisions, with standard electrosurgical blade incisions taking the longest to heal. Wound strength testing supports the wound healing prediction in that scalpel incisions heal the fastest. ACE incisions were equal in pull strength to the scalpel incisions at 2 weeks and continued to heal as well as the scalpel incisions through the six-week endpoint. Standard electrosurgical blade incisions were the same as ACE incisions at one week, but weaker than ACE and scalpel incisions at each subsequent week.

Often the most important surgical result beyond the therapeutic intent of the surgery is how the scar appears to the patient. Patients are not able to see what was done surgically, but they can see the entry point. Cosmetic results in this study were judged by a panel of 12 surgical experts. The endpoint was based on 6-week healing of the various incisions. Experts were blinded to the assignment for each incision and asked to grade them from best to worst. Based on the results, incisions made by scalpel and ACE were indistinguishable. Scalpel rated as better or best of the three 85% of the time, and ACE rated as better or best 98% of the time. Both scalpel and ACE were significantly superior to standard electrosurgery.

While electrosurgery has become an essential surgical technology, historically there has been a reluctance to make cutaneous incisions. This stems from concerns about excessive scaring, delayed healing and increased infection compared to cold scalpel incisions. Modern electrosurgical generators can produce distinct radio frequency waveforms each having a different surgical effect. Advances in electronics allow the production of a pure sinusoidal “cut” waveform. In contrast to the “coagulate” modes, the cut mode allows tissue cleavage with minimal thermal damage to surrounding tissue. Early studies, using what today would be considered outdated electrosurgical technology, revealed concerns about wound healing after skin incision using electrosurgery [4,7,12].These concerns were focused on delayed healing, excessive scaring and potential for increased infection [13-15]. With the advent of modern electronics, wave modulation has evolved to include a pure sinusoidal format able to vaporize tissue with minimal collateral thermal damage. Even so, some bias against using electrosurgery for skin incisions continues to be propagated today [4,7,12].

More recent studies using modern electrosurgical generators able to produce a pure sinusoidal cut waveform have demonstrated that concerns regarding the use of electrosurgery for skin incisions are unfounded [8,9,11,16-23]. While there were no wound healing complications in this study, the additional thermal damage from a pure sinusoidal cut wave was enough to delay healing of the standard electrosurgical blade incisions when compared to ACE incisions. Geometric Electron Modulation (GEM) Technology achieves a scalpellike cutting effect by creating low voltage plasma. This technology optimizes the voltage for the ACE blade geometry and modulates power based on tissue impedance using ACE mode on the MegaPower generator. It focuses energy to the tapered edges of the ACE blade and delivers the minimum power required to cut the tissue.

Previous studies support the use of standard electrosurgical blades for skin incisions. The results of this study demonstrate that incisions made using ACE heal better than standard electrosurgical blade incisions and similar to scalpel incisions. At 6 weeks, there was no substantial difference in wound strength and cosmetic wound healing between scalpel and ACE incisions.

New technologies such as ACE have decreased or eliminated the tissue effect gap between electrosurgical incisions and scalpel incisions. Beyond these cosmetic results, ACE has the potential to save surgeon time by eliminating the need to pass scalpels and standard electrosurgical blades back and forth. By eliminating the constant hand-off of the scalpel, the risk of sharps injuries is also reduced.

In conclusion, ACE represents a significant improvement in electrosurgical technology for skin incisions. The results of this study demonstrate that incisions made using ACE heal better than standard electrosurgical blade incisions and similarly to scalpel incisions. The combination of good cosmetic results, time savings, and sharps safety makes MEGADYNE ACE BLADE™ 700 Soft Tissue Dissector a viable alternative to the scalpel.

## Figures and Tables

**Figure 3a.**
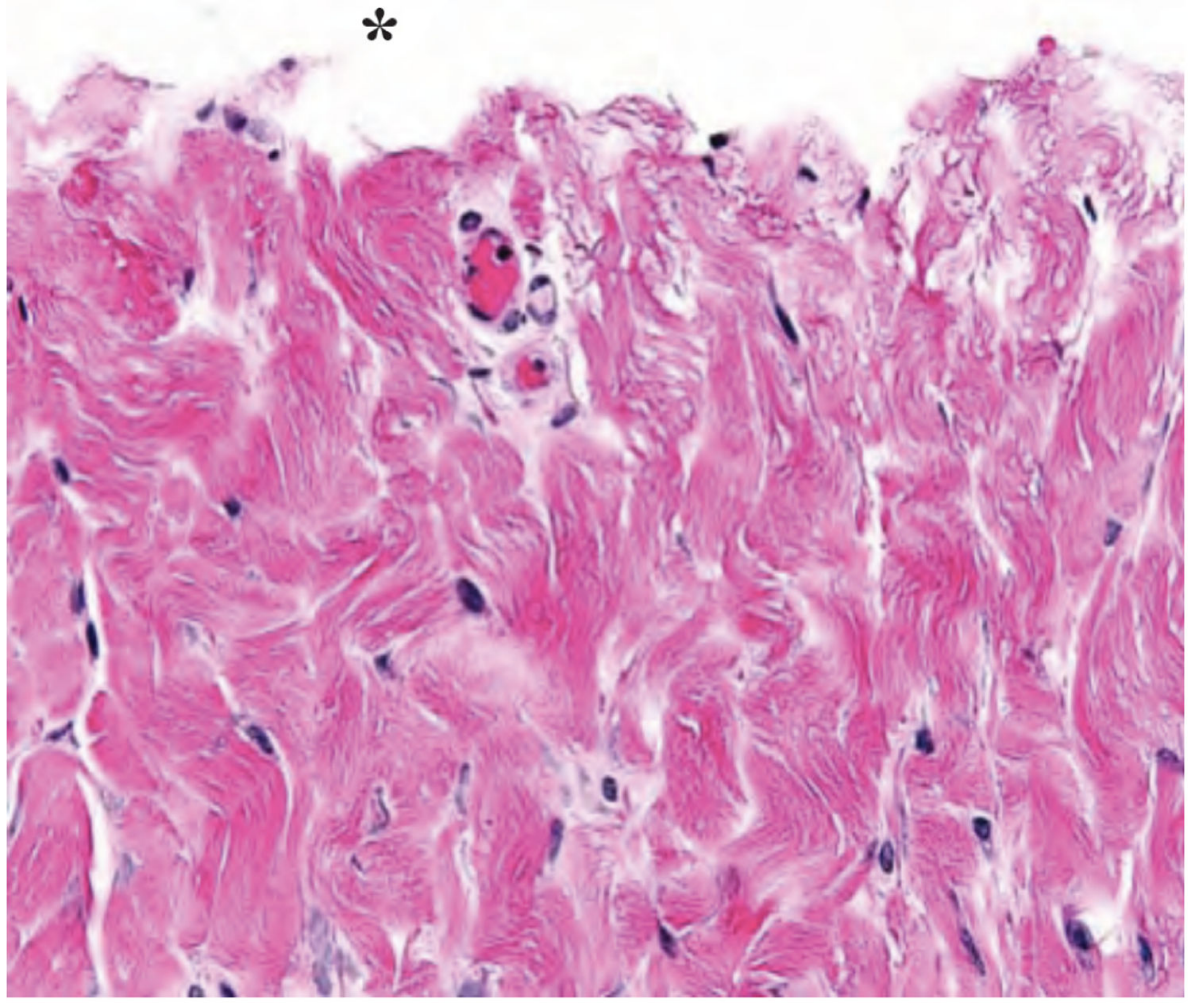
Scalpel incision time 0 histopathology of the dermis. Note the lack of thermal damage on the cut surface (indicated by an asterisk).

**Figure 3b.**
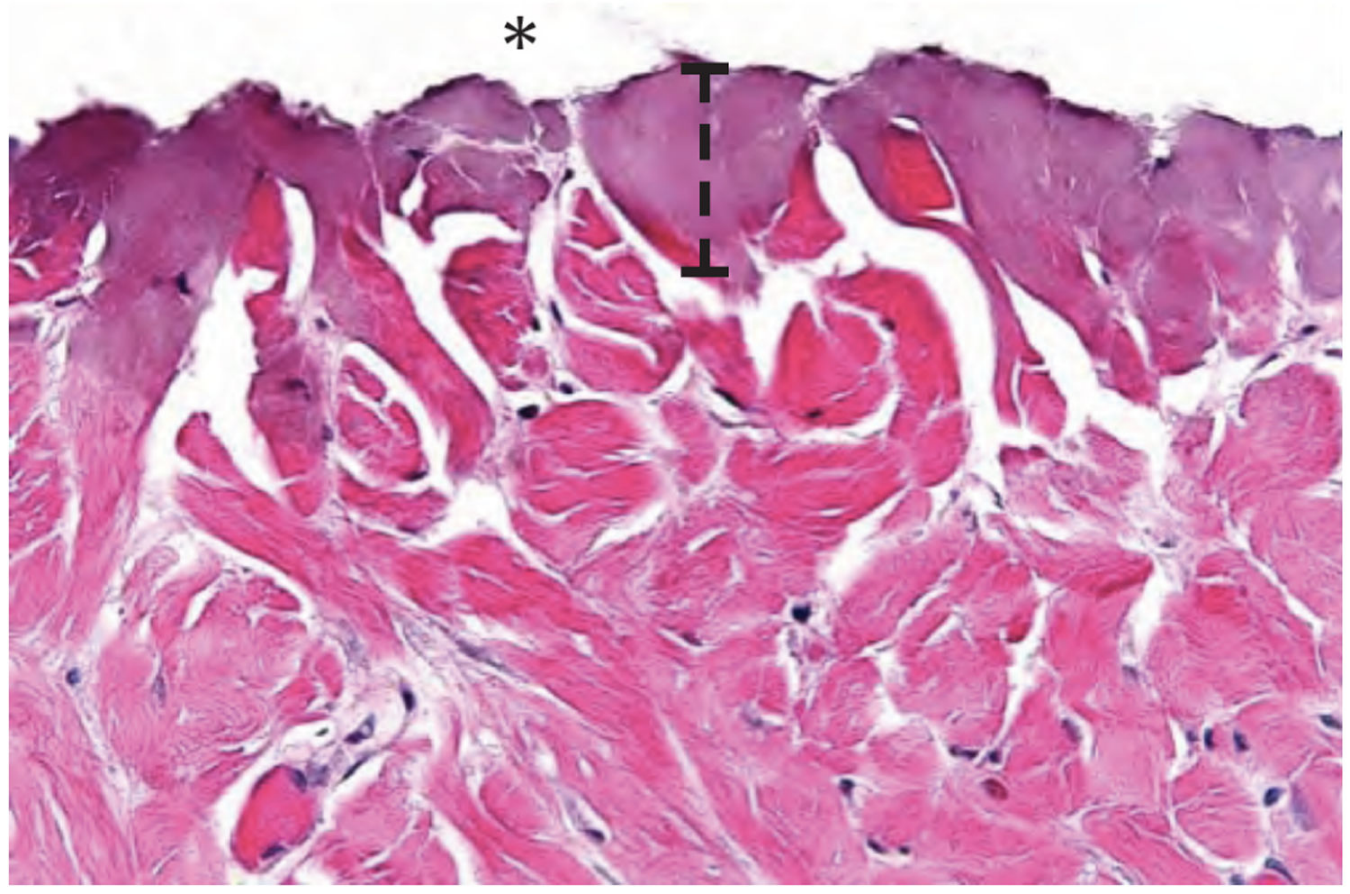
ACE incision time 0 histopathology of the cut surface of the dermis (indicated by an asterisk). Note the width of thermal damage (indicated by bracket).

**Figure 3c.**
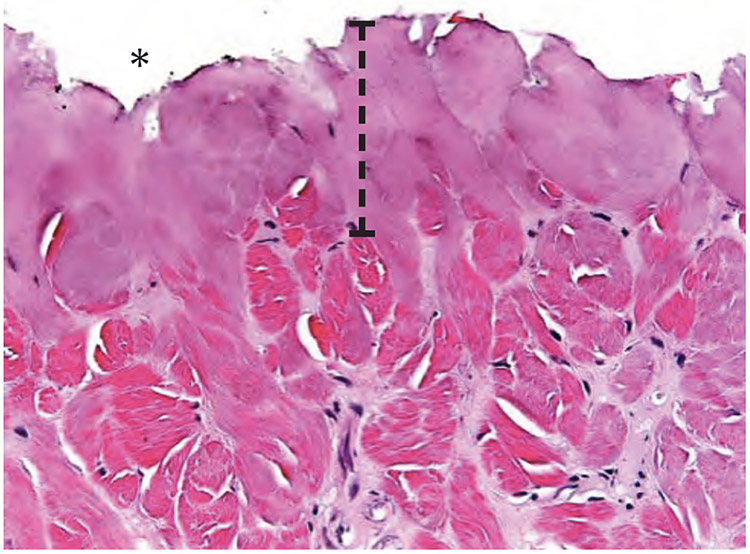
Standard electrosurgical cut incision time 0 histopathology of the cut surface of the dermis (indicated by an asterisk). Note the width of thermal damage (indicated by bracket) is approximately 2.5x larger than seen with ACE incision.

**Table 1.** Summary of measurements

	Scalpel	ACE Blade	Standard ES
**Dermal Scar Tissue Width**			
Week 0	0.00 mm	0.12 mm	0.26 mm
Week 2	0.15 mm	0.19 mm	0.25 mm
Week 3	0.46 mm	1.20 mm	1.70 mm
Week 6	1.00 mm	0.60 mm	2.70 mm
**Wound Strength**			
Week 1	4.9 lbf	2.1 lbf	2.2 lbf
Week 2	11.5 lbf	10.6 lbf	7.8 lbf
Week 3	12.8 lbf	12.7 lbf	11.1 lbf
Week 6	35.6 lbf	40.3 lbf	22.3 lbf
**Cosmetic Wound Healing**			
Best	58%	42%	0%
Middle	27%	56%	17%
Worst	15%	2%	83%
